# Survival prediction from clinico-genomic models - a comparative study

**DOI:** 10.1186/1471-2105-10-413

**Published:** 2009-12-13

**Authors:** Hege M Bøvelstad, Ståle Nygård, Ørnulf Borgan

**Affiliations:** 1 Department of Mathematics, University of Oslo, PO Box 1053 Blindern, NO-0316 Oslo, Norway; 2 Norwegian Computing Center, PO Box 114 Blindern, NO-0314 Oslo, Norway

## Abstract

**Background:**

Survival prediction from high-dimensional genomic data is an active field in today's medical research. Most of the proposed prediction methods make use of genomic data alone without considering established clinical covariates that often are available and known to have predictive value. Recent studies suggest that combining clinical and genomic information may improve predictions, but there is a lack of systematic studies on the topic. Also, for the widely used Cox regression model, it is not obvious how to handle such combined models.

**Results:**

We propose a way to combine classical clinical covariates with genomic data in a clinico-genomic prediction model based on the Cox regression model. The prediction model is obtained by a simultaneous use of both types of covariates, but applying dimension reduction only to the high-dimensional genomic variables. We describe how this can be done for seven well-known prediction methods: variable selection, unsupervised and supervised principal components regression and partial least squares regression, ridge regression, and the lasso. We further perform a systematic comparison of the performance of prediction models using clinical covariates only, genomic data only, or a combination of the two. The comparison is done using three survival data sets containing both clinical information and microarray gene expression data. Matlab code for the clinico-genomic prediction methods is available at http://www.med.uio.no/imb/stat/bmms/software/clinico-genomic/.

**Conclusions:**

Based on our three data sets, the comparison shows that established clinical covariates will often lead to better predictions than what can be obtained from genomic data alone. In the cases where the genomic models are better than the clinical, ridge regression is used for dimension reduction. We also find that the clinico-genomic models tend to outperform the models based on only genomic data. Further, clinico-genomic models and the use of ridge regression gives for all three data sets better predictions than models based on the clinical covariates alone.

## Background

Predicting the outcome of a disease or some disease related phenotype based on microarrays or other high-throughput data is an important application of genomic data. One particular instance of this problem is the prediction of the time to some disease specific event like death or relapse, often referred to by the technical term survival time or failure time. The most widely used model for survival data is the Cox proportional hazards model [1] which describes the instantaneous risk of failure at time *t *by the hazard rate(1)

Here **x **= (*x*_1_, ..., *x*_*p*_)^*T *^is a set of genomic variables, e.g. gene expression or snp measurements, ***β ***= (*β*_1_, ..., *β*_*p*_)^*T *^is a vector of regression coefficients describing the effects of each variable, and *h*_0_(*t*) is the baseline hazard giving the hazard rate of an individual with all *x*_*j *_equal to zero. In the sequel we will refer to (1) as the *genomic model*.

A major challenge of high-dimensional genomic data, where the number *p *of predictors is much larger than the number of individuals *n *(*p *>>*n*), is the problem of overfitting. By using complex enough models, there are infinitely many parameter combinations that fit the data perfectly, but these will make use of random predictor-response correlations, resulting in poor predictions on external data sets. The solution is to use some form of dimension reduction, or regularization, on the variable space to obtain a more parsimonious model. In the classical case of ordinary linear regression there are many methods for such high-dimensional data, including variable subset selection methods, principal components regression (PCR), partial least squares (PLS), ridge regression, and the lasso; see e.g. [2] for a review. All these high-dimensional prediction methods have been adapted to the Cox regression setting for censored survival data, e.g. [3] and [4] combining univariate selection and PCR, [5] applying PLS, [6] using ridge regression, and [7] and [8] applying the lasso. In Bøvelstad *et al*. [9] a thorough comparison of the prediction performance of these methods was performed using three well known high-dimensional microarray gene expression data sets.

Together with the genomic data, information on demographic and clinical variables (or covariates) often exists. Examples of such variables are age, stage, grade, tumor thickness, and lymph node status. The clinical covariates may be important predictors known to be correlated to survival. Also, there exist many established prognostic indices that are combinations of such classical clinical covariates and that are widely used, like e.g. the Nottingham Prognostic Index (NPI) [10] used for predictions in breast cancer or the International Prognostic Index (IPI) [11] for predicting survival of patients with non-Hodgkin's lymphoma. Specifically, assume that we have a vector **z **= (*z*_1_, ..., *z*_*q*_)^*T *^of demographic and clinical covariates. A prediction model using only these covariates can be obtained using the Cox model(2)

where ***γ ***= (*γ *_1_, ..., *γ*_*q*_)^*T *^is a vector of regression coefficients for the demographic and clinical variables. We will in the sequel refer to (2) as the *clinical model*.

Even though clinical and demographic variables have a prognostic value, predictions based on such covariates may not be accurate enough. For this reason, an immense effort has been put into finding genomic variables that can contribute to better predictions and hence more tailored treatment schemes, e.g. [12-14]. The hope has been that the genomic variables would fully replace the information obtained from the clinical and demographic variables. As a consequence, clinical and demographic variables with known predictive value have not been taken into consideration when building prediction models from genomic data. However, some studies (e.g. [15]) have shown that established clinical predictors are not outperformed by genomic variables as prediction tools. It may hence be useful to also consider established clinical covariates when building prediction models.

Recently many authors have started focusing on combining clinical and demographic variables with genomic data forming what has been called clinico-genomic models. This has been done mostly for classification of patients, e.g. into high-risk and low-risk groups [15-17]. Clinico-genomic models for survival prediction using the Cox model [18-22] or Bayesian Weibull tree models [23] have also been proposed. Common to these papers is that they find that the clinico-genomic models seem to outperform the models using either clinical covariates alone or genomic covariates alone. Combining such data in a Cox model would yield a *clinico-genomic model *given by(3)

where **z **are the clinical and demographic covariates and **x **the genomic variables. Assuming that the established low-dimensional clinical and demographic covariates are known to have effect on survival, it is natural to perform dimension reduction only on the high-dimensional genomic covariates. Combining clinical information and high-dimensional genomic data in a Cox model is, however, not straightforward. In this paper we show how this can be done for seven well-known prediction methods based on the Cox model, namely univariate selection, unsupervised and supervised principal components regression and partial least squares regression, ridge regression, and the lasso. Many of these methods have been used with success when predicting survival using only genomic data, but have to our knowledge not been systematically studied for the combined clinical and genomic data.

The objectives of this paper are (i) to make a systematic comparison of the performance of the seven prediction methods when using both clinical covariates and genomic variables, and (ii) to compare the overall prediction performance of the clinical model (2), the genomic model (1), and the clinico-genomic model (3). The comparison will be performed using three survival data sets containing both clinical information and microarray gene expression data.

## Methods

We assume that the demographic and clinical covariates **z **are known to have an effect on survival (but with unknown size of the effect). Thus, for the model (2) with only clinical covariates no variable selection or dimension reduction will be done. We simply fit an ordinary Cox model to the data to obtain parameter estimates.

Bøvelstad *et al*. [9] described how univariate selection, PCR, supervised PCR, PLS, ridge regression, and the lasso can be applied to model (1) using the genomic variables as the only covariates. The same is described for supervised PLS in Nygård *et al*. [5]. The methods are similar to the corresponding ones described below for the clinico-genomic setting.

When we have both clinical covariates and genomic variables, we will treat the clinical model (2) as a starting model. The additional effects of the genomic variables **x **are found by simultaneously estimating the effects of **x **and **z **using (3), but where the dimension reduction is applied only to **x**. In the next subsections we describe in more detail how this can be done for the prediction methods under study. All seven methods assume a given model complexity, represented by a parameter *λ*. The optimal value of *λ *can be found using cross-validation, which will be described later.

### Prediction methods for clinico-genomic models

#### Univariate selection

We start out with the clinical model (2), i.e. a Cox regression model including only the demographic/clinical covariates **z**. For each gene *g*, we test this model versus a Cox model including the gene together with the clinical variables, i.e. we test *h*(*t*∣**z**, *x*_*g*_) = *h*_0_(*t*) exp(**z**^*T *^***γ ***+ *β*_*g*_*x*_*g*_) versus *h*(*t*∣**z**) = *h*_0_(*t*) exp(**z**^*T *^***γ***). These tests are performed using a local score test [[24], Chapter 8.5]. The *λ *top ranked genes from the models with the smallest *P*-values are picked out and included along with the clinical covariates **z **in a multivariate Cox regression model.

#### Principal components regression (PCR)

Principal components analysis (PCA) finds linear combinations of the genomic variables, where each new linear combination has maximal variance under the constraint of being orthogonal to the first ones. We find the *λ *first principal components using PCA on the genomic variables **x**. We then include the principal components together with the demographic and clinical covariates **z **in a multivariate Cox regression model.

#### Supervised principal components regression

Since the principal components are constructed without considering the response, there is no guarantee that the components are associated with patient survival. With this argument, [3] and [4] proposed a supervised PCR, where a pre-selection of genes significantly correlated to survival is included before the PCA is applied. Following this approach, we first pick out *λ*_1 _percent of the top ranked genes using univariate selection as described above. We then apply PCA to this subset of genes and include *λ*_2 _of the first components together with **z **in a multivariate Cox model.

#### Partial least squares (PLS) regression

Like PCR, partial least squares regression is based on linear combinations of the genomic variables. However, PLS uses combinations that are correlated with survival. There are many suggestions on how to perform PLS for the Cox regression setting. We will use the method of Nygård *et al*. [5] that allows for inclusion of demographic and clinical covariates together with the genomic variables, but only performs dimension reduction on the latter.

#### Supervised partial least squares regression

PLS finds linear combinations in the space of the genomic variables, which have the property of maximizing the covariance between the components and the response (see e.g. [25]). The covariance is the product of the variance of the components and the correlation between the components and the response. It is often experienced that the variance part is dominating, causing PLS to behave very much the same way as PCR. As for PCR, it can therefore be argued that also PLS may benefit from a preselection step finding the genes most correlated to patient survival. In our supervised PLS Cox method for both genomic and demographic/clinical variables we use the same algorithm as in the supervised PCR method described above, except that the PCR step on the pre-selected genes is replaced by the PLS algorithm given in [5].

#### Ridge regression

Ridge regression [26] shrinks the regression coefficients by imposing a penalty on their squared values. Van Houwelingen *et al*. [6] showed how ridge regression can be applied to the Cox regression setting with high-dimensional genomic data by maximizing a penalized log-likelihood. We may extend the approach in [6] by including lower-dimensional covariates **z **in the log-likelihood, but performing penalization only on the high-dimensional covariates **x**. This gives us the following penalized log-likelihood:

where *l*_full _(***λ***, ***β***, *H*_0_) is the full log-likelihood given by [24]

Here, *t*_*i *_denotes the possibly censored survival time of individual *i*, and *d*_*i *_indicates whether this survival time is observed (*d*_*i *_= 1) or censored (*d*_*i *_= 0). Further, *H*_0 _(*t*) is the cumulative baseline hazard and Δ*H*_0_(*t*_*i*_) is its increment at time *t*_*i*_.

To reduce the computational burden, we use the approach of van Houwelingen *et al*. [6] to obtain parameter estimates. They noted that the estimating equation ∂*l*_pen_(***γ***, ***β***, *H*_0_)/∂***β ***= **0 **implies that the resulting estimate for ***β ***lies in the space spanned by the columns of **X**, where **X **is the *n *× *p *matrix whose *i*th row is the vector  of genomic variables for patient *i*. Therefore, we may write ***β ***= **X**^*T*^***ψ ***, for some ***ψ ***. The dimension of the problem is thus reduced from *p *to *n*. In terms of ***ψ ***, we have

where **u**_*i *_is the *i*^th ^row of **U **= **XX**^*T*^.

#### Lasso

The lasso [27,28] shrinks the regression coefficients in a similar manner as ridge regression, but uses the absolute values instead of the squared values. Penalizing the absolute values has the effect that a number of the estimated coefficients will become exactly zero, which means that the lasso is also a variable selection method. Like ridge regression, the lasso can be modified to include clinical and demographic covariates with penalization only of the high-dimensional genomic variables. More precisely, the Cox regression coefficients in the clinico-genomic model (3) can be found by maximizing *l*(***γ***, ***β***) - . Here, *l*(***γ***, ***β***) is the logarithm of the Cox partial likelihood for model (3) given by

where *R*(*t*_*i*_) is the risk set of time *t*_*i*_. We have used the lasso implementation of Cox regression due to Park and Hastie [8], available through the R package glmpath. The clinical covariates were specified using the "nopenalty.subset" argument.

### Cross-validation

All methods described in the previous subsections depend on a parameter *λ*, representing the complexity of the genomic part of the model: the number of genomic variables for univariate selection, the number of linear components for PCR and PLS, and the penalty parameter for ridge regression and the lasso. For supervised PCR and supervised PLS the model complexity depends on both the number of genomic variables and the number of PCR/PLS components, i.e. *λ *= (*λ *_1_, *λ *_2_) is two-dimensional for these two methods.

The value of *λ *must be estimated, and finding the optimal model complexity is a difficult but crucial task when analyzing high-dimensional data. The method of cross-validation (CV) can be used to find the optimal model complexity *λ*. We will use 10-fold CV together with Verweij and van Houwelingen's [29] CV criterion, which is based on the Cox log partial likelihood. After having found the optimal *λ *for the genomic part of the model, this value is used in a prediction method as described in the previous subsections to find estimates of ***β ***for the genomic model (1), and estimates of ***γ ***and ***β ***for the clinico-genomic model (3).

### Prediction performance

Thus far we have described how to find estimates of ***β ***for the genomic model (1) and estimates of ***γ ***and ***β ***for the clinico-genomic model (3). To evaluate how good these estimates are for prediction, we will follow the evaluation scheme proposed in Bøvelstad *et al*. [9]. More precisely, we will compare the prediction performance of the seven methods using the following approach: The data are randomly split into training and test sets, where the training set is about twice as large as the test set. Then 10-fold CV is used on the training set to find an estimate  of the optimal model complexity for the genomic part of the model. Given , we use the whole training set to obtain an estimate  for the effects of the genomic covariates in model (1), and similarly for model (3) the estimates  and  for the effects of the demographic/clinical and genomic covariates, respectively. For the clinical model (2),  is estimated directly by ordinary Cox regression using the whole training set since no variable selection or dimension reduction is performed on these covariates. Note that the test data are set aside in the whole model building procedure, and are only used to evaluate the final prediction model. This is done in order to ensure a completely independent evaluation.

As a measure of how well a prediction model performs on the test data set, we will use the difference in deviance between a fitted model and the null model containing no covariates. Specifically, for the clinico-genomic model this difference in deviance is given by(4)

where *l*^(test) ^() and *l*^(test) ^(**0**) are the Cox log partial likelihood for the test data evaluated at ()^*T *^and **0**, respectively. The difference in deviances for the clinical model and the genomic model can also be found using (4), but where *l*^(test) ^() is replaced by *l*^(test) ^() and *l*^(test) ^(), respectively. Note that 1 - , where *m *is the number of subjects in the test data set, may be interpreted as a measure of the variation in the test data explained by the prediction model [30]. The performance of a model is good when the difference in deviance is small.

Bøvelstad *et al*. [9] showed that the relative performance between the prediction methods could depend on the particular training/test splits. To ensure a fair comparison, we therefore follow their approach and generate 50 random splits of the data into 2:1 training and test sets. The performance of the methods are then evaluated by the median and the spread of the difference in deviance over the 50 splits.

## Results

Three different data sets will be used in order to compare the performance of the prediction methods described in the Methods section, as well as the performance of the clinical models, the genomic models, and the clinico-genomic models. The data sets are described below, along with the results.

### Breast cancer data

The first data set is from the paper of van Houwelingen *et al*. [6] and contains 4919 gene expression measurements, clinical covariates, and censored survival times from 295 Dutch women diagnosed with breast cancer. The data have been visited in a number of papers, and is a modified version of the data introduced in the papers of van't Veer *et al*. [12] and van de Vijver *et al*. [31]. The median follow-up time is 7.2 years, and out of the 295 patients 27% experienced breast cancer death. As clinical covariates, we use tumor diameter (mm), lymph node status (positive/negative), and grade (good/intermediate/poor), which are classical clinical covariates used for prediction of breast cancer survival. Also, the classification rule that defines the Nottingham Prognostic Index (NPI) [10] is based on these three covariates. For more information on the data, see [6].

**Figure 1:**
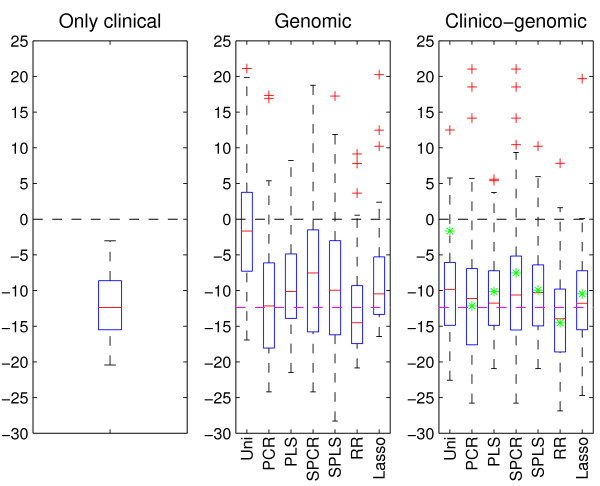
**Breast cancer data**. Results after applying the clinical model (left-hand boxplot) and the seven prediction methods to both the microarray gene expression data (center boxplot) and the combined data (right-hand boxplot). In all three boxplots, the horizontal line at zero indicates the null model with no covariate information. For the center and right-hand boxplots, the dashed magenta line indicates the median of the clinical model. Further, the green stars in the right-hand boxplot are the median of each of the seven methods when applied to the microarray gene expression data. A small value of the difference in deviance corresponds to a good prediction performance. Uni - univariate selection, PCR - principal components regression, PLS - partial least squares, SPCR - supervised PCR, SPLS - supervised PLS, and RR - ridge regression.

The results when applying the described methods to the data are summarized in the boxplots of Figure 1. The data are divided 50 times at random into training and test sets containing 200 and 95 patients, respectively. As in the paper of Bøvelstad *et al*. [9] we will for each method consider the median of the 50 values of difference in deviance as the measure of main interest. For all three boxplots, the horizontal black line at zero indicates the null model (with no covariate information included), and is displayed for reference.

The left-hand boxplot of Figure 1 shows the difference in deviance obtained from applying the clinical model to the 50 training/test splits. The median of these 50 values is further displayed, for easy comparison, by a dashed magenta line in the other two boxplots.

The center boxplot of Figure 1 displays the predictions made from the seven methods when using the microarray gene expression data as covariates. From the plot we see that univariate selection has the poorest performance, and that many of its 50 predictions are worse than what can be obtained using a prediction model with no covariate information. From the plot we also see that PLS, supervised PLS, supervised PCR, and the lasso give poorer predictions than the clinical model. PCR has on the median a similar performance as the clinical model, but predictions using PCR yields larger variation. Ridge regression is the method with the best prediction performance according to our definition, and also the method with the smallest variation. Also, ridge regression is the only method able to improve predictions using genomic information compared to using the clinical model (2).

Finally, the right-hand boxplot of Figure 1 displays the results when using both types of data for prediction. For comparison purposes, we have indicated the median of each of the seven methods when applied to the microarray gene expression data by a green star. Studying the plot, we see that the methods have a more similar performance and are less variable than the corresponding results from the genomic model. Again, univariate selection has the poorest performance, and ridge regression is the only method able to make predictions that are better than when using clinical data alone. Compared to the genomic model, all methods except PCR and ridge regression have improved prediction when using both clinical and genomic covariates.

### DLBCL data

The second data set, introduced in Rosenwald *et al*. [32], consists of censored survival times and 7399 microarray gene expression measurements for 240 patients with diffuse large-B-cell lymphoma (DLBCL). The median follow-up time is 2.8 years, and 57% of the patients died during follow-up. For 222 of these patients, we also have information on the International Prognostic Index (IPI), which is a well-established prognostic score derived from five clinical covariates (see [11] for more details). The IPI has levels low, medium, and high. Since we want to compare models containing both types of data, we will restrict our attention to the smaller set of patients. For more information on the data, see [32].

**Figure 2:**
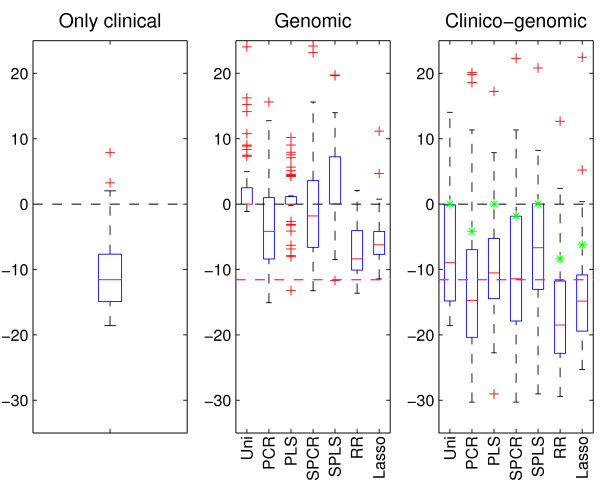
**DLBCL data**. Results after applying the clinical model (left-hand boxplot) and the seven prediction methods to both the microarray gene expression data (center boxplot) and the combined data (right-hand boxplot). Further details of the plot are given in the legend of Figure 1.

Figure 2 shows the results after applying the various models and methods to the 50 random training (150 patients) and test (72 patients) splits of the data. From the center boxplot, it is clear that when using only microarray gene expression data, all methods have a rather poor performance. In fact, none of the methods are able to make predictions that are better than when using the clinical covariate IPI alone, and many have as poor as or poorer performance than if using the null model. As for the breast cancer data, ridge regression has the best prediction performance among the seven methods. Investigating the right-hand boxplot, we see that using the clinico-genomic model yields a vast improvement in prediction performance for all methods. Again, ridge regression has the best performance and is able to obtain improved predictions compared to the clinical model. The latter is also the case for the lasso and PCR.

### Neuroblastoma data

The last data set is from Oberthür *et al*. [33] and consists of 362 patients suffering from neuroblastoma. For each patient, we have information on their risk group according to the current German neuroblastoma trial (NB2004, levels low/intermediate/high) as well as 9978 microarray gene expression values and its (possibly) censored survival time. Median follow-up time for the patients are 3.8 years, and out of the 362 patients 21% died from the disease. The patients were introduced in [33] as two different sets; one "training set" of 256 patients and one "test set" of 120 patients. We merged the two, and the 9978 microarray gene expression measurements are from probes shared by both sets. Due to few events in the two lower NB2004 risk groups, we chose to combine them into one group. Also, 14 patients were omitted from our study due to missing clinical information. For more information on the data, consult [33].

**Figure 3:**
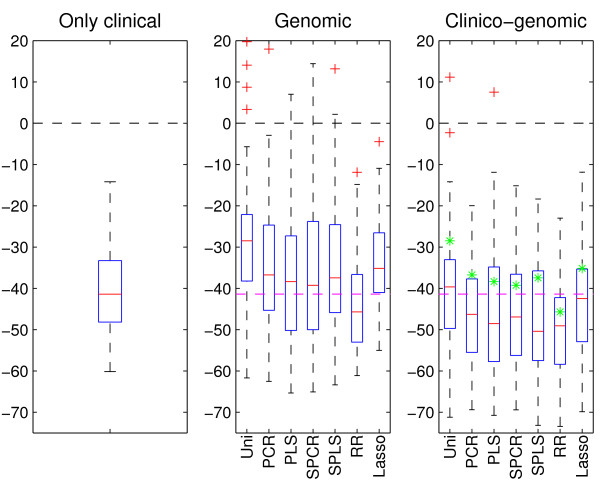
**Neuroblastoma data**. Results after applying the clinical model (left-hand boxplot) and the seven prediction methods to both the microarray gene expression data (center boxplot) and the combined data (right-hand boxplot). Further details of the plot are given in the legend of Figure 1.

We generated 50 random splits of training (240 patients) and test (122 patients) sets from the data, and formed boxplots from the results which are displayed in Figure 3. From the center plot, we observe that when using microarray gene expression data, only ridge regression is able to make predictions that are better than if using only the NB2004 stratification index. This is in accordance with the observations made for the breast cancer data. As seen for the DLBCL data, combining the clinical covariate and the microarray gene expression data resulted in a large improvement in prediction ability for all prediction methods. In fact, all methods but univariate selection are able to make better predictions than the clinical model using the NB2004 strata alone. For the clinico-genomic models, supervised PLS has the best median performance, whereas ridge regression has the second best performance.

### Results summary

**Figure 4:**
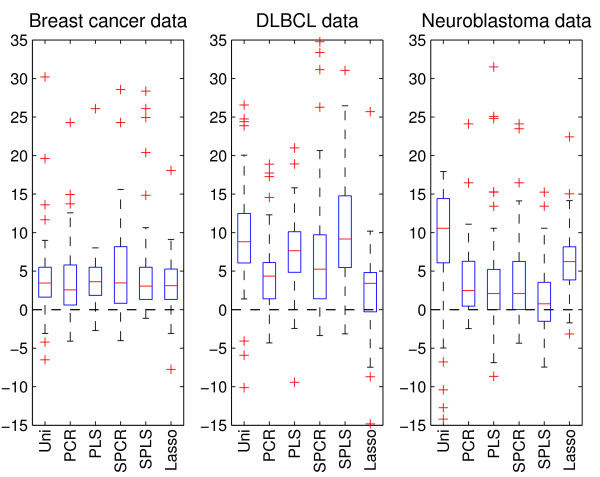
**Difference in deviance from the ridge model**. The boxplots give the difference in deviance between the six methods and ridge regression when using the combined data in a clinico-genomic model. The plots thus give a pairwise comparison between ridge regression and the other methods, in addition to giving an illustration of the variation due to regression methods corrected for the variation due to the 50 random training/test splits. For method abbreviations, see Figure 1.

Observing each boxplot in Figures 1, 2, 3, there is a fairly large spread in the difference in deviance over the 50 splits. This is partly due to variation caused by splitting the data at random into training and test sets, and partly due to variation in the performance of the prediction methods for the various splits. In order to explore how much of the variation that is due to the latter when using the combined data in a clinico-genomic model, we use ridge regression as a benchmark and, for each of the splits, compute the difference between the deviance of the six other methods and the deviance of ridge regression. Figure 4 shows boxplots of these differences for the 50 splits, and represents a pairwise comparison between ridge regression and the other methods when applied to the combined data. The figure shows that for a majority of the splits, ridge regression has a better prediction performance than all the other methods on all three data sets. Note that this also applies for the neuroblastoma data where supervised PLS had better median performance than ridge regression (right-hand plot of Figure 3).

## Discussion and conclusions

In treatment of patients with cancer and other fatal diseases, obtaining accurate survival predictions is a crucial step for better treatment decisions and prolonged survival. Several recent studies [15-17,19-23] have suggested that combining clinical information and genomic data may lead to better predictions than when using such data separately. However, for the well-known Cox regression model, combining low-dimensional clinical data with high-dimensional genomic data is not straightforward. We have shown how this can be done for seven well-known prediction methods used for high-dimensional data. In addition, we have performed a systematic study in order to (i) study the behavior of these seven methods when applied to the combined data, and (ii) compare the survival predictions obtained when using only clinical data, only genomic data, or a combination of the two.

To compare the prediction methods, we used the comparison scheme of Bøvelstad *et al*. [9]. The scheme was applied to three survival data sets containing both clinical information and microarray gene expression measurements for patients diagnosed with breast cancer [6,12,31], diffuse large-B-cell lymphoma (DLBCL) [32], and neuroblastoma [33]. In our study, we have assumed that the clinical covariates are known to have an effect on survival, so no selection or dimension reduction have been applied to these covariates. For multiple pairs of training/test sets we employed cross-validation on the training sets to find the optimal complexity of the genomic part of the models, and evaluated the models on the independent test sets. Doing this, we did not risk the danger of getting overly optimistic results for the genomic predictor, which some earlier studies have shown. In van't Veer *et al*. [12], for example, the genomic predictor was both derived and evaluated on the same data, leading to a heavily overestimated prediction strength for this predictor. This was criticized by Tibshirani and Efron [34], who proposed the method of *K*-fold pre-validation (see also [35]), where the prediction for each individual *i *is based on a rule made with fold *g*, *i *∈* g*, left out. The pre-validation procedure is especially suited when data are sparse, as it also uses the training data in the evaluation procedure. Bøvelstad *et al*.'s [9] procedure of fitting and evaluating the methods on multiple random splits into training and test sets is an alternative way of utilizing the whole data set in the evaluation procedure.

We find that ridge regression has the best median prediction performance for the breast cancer data and the DLBCL data, and has the second best performance for the neuroblastoma data (Figures 1, 2, 3). However, in the pairwise comparison (Figure 4), ridge regression performs better on all three data sets than all the other methods for more than half of the 50 splits studied. For the breast cancer data and the DLBCL data, comparing the unsupervised versions of PCR/PLS with the supervised versions for the clinico-genomic models indicate that pre-selection of genes is not improving predictions, and rather giving more unstable results. The lasso, which can be thought of as a selection method, has a rather poor performance in two out of three data sets. Simple univariate selection has the poorest performance of the methods studied. This is evident in all three data sets. Its performance is fairly good on the combined data, but this is simply because it for most of the 50 splits selects no genes, and thus behaves more or less as the clinical model.

The second goal of our comparative study was to investigate the prediction performance of models that utilize only clinical data, only genomic data, or a combination of the two. Based on the three data sets, our comparison study indicated that using genomic data alone may lead to poorer predictions than what can be obtained from established clinical predictors. In the cases where the genomic models were better than the clinical ones, ridge regression was used for dimension reduction. We also found that the clinico-genomic models tend to outperform the models based on genomic data alone. However, the improvement of using combined data varied among different diseases. In our study there was a difference between the breast cancer data on one hand, and the DLBCL and neuroblastoma data on the other. For the breast cancer data set, the clinical covariates and the genomic covariates seemed to contain much of the same information for the purpose of prediction. Thus, the predictions made using microarray gene expression data alone did not differ much from the predictions made when using both the clinical data and the microarray gene expression measurements, as observed in Figure 1. This is in agreement with the results found in [18]. For the DLBCL and the neuroblastoma data sets, the information was more orthogonal and large improvements were made when combining the data into clinico-genomic models (Figures 2 and 3).

We conclude that combining traditional clinical covariates with high-dimensional genomic data may lead to better predictions than what can be achieved using the data separately. Also, the results from the three data sets studied indicate that the choice of high-dimensional prediction method may be important. Ridge regression seems to be the method that most often achieves the best predictions when applied to both the genomic model and the clinico-genomic model. However, we emphasize that additional studies investigating more data sets, as was done in [18], should be carried out in order to confirm our findings and draw final conclusions.

Finally we want to point out that the purpose of this paper has been to perform a methodological study comparing the seven methods for building prediction models using clinical and genomic data. This is different from finding a prediction model for a given data set. In order to build a clinico-genomic prediction model using a given dimension reduction method, one should use the whole data set (no test data is set aside for validation) and proceed as described for a single set of training data in the Methods section.

## Authors' contributions

The project idea emerged from our study of prediction methods based on genomic data [9]. HMB and SN developed and implemented the Matlab code for the various prediction methods. HMB organized the data sets and performed the computations. HMB and SN drafted the paper. ØB discussed the project with HMB and SN as it progressed and commented on various drafts of the manuscript. All authors have read and approved this manuscript.
